# China's Modified Version of Sniffin' Sticks 12-Identification Test Used in Chinese Parkinson's Disease and Multiple System Patients: Comparison of Three Olfactory Testing Methods

**DOI:** 10.1155/2024/3561881

**Published:** 2024-06-25

**Authors:** Huijing Liu, Wei Du, Shuhua Li, Ying Jin, Haibo Chen, Wen Su

**Affiliations:** Neurology Department Beijing Hospital National Center of Gerontology Institute of Geriatric Medicine Chinese Academy of Medical Sciences, No. 1 Dahua Road, Dong Dan, Beijing 100730, China

## Abstract

**Objectives:**

The aim of this study was to compare the Sniffin' Sticks 12-identification test (SIT-12), China-modified version of the SIT-12 test (Ch-SIT-12) and brief smell identification test for Chinese (B-SITC) in Chinese population of Parkinson's disease (PD) and multiple system atrophy (MSA).

**Methods:**

36 patients with PD and 7 patients with MSA were enrolled in this study. Three olfactory testing methods (SIT-12, Ch-SIT-12, and B-SITC) were used to test the olfactory function in all participants. Furthermore, demographic and clinical data were collected.

**Results:**

There was no significant difference between three olfactory tests in patients with PD (B-SITC vs. SIT-12: *P*=0.508; Ch-SIT-12 vs. B-SITC: *P*=0.146; and SIT-12 vs. Ch-SIT-12: *P*=0.375). Tremor-dominant (TD) subtypes have better olfactory function than akinetic-rigid dominant (ARD) subtypes when using Ch-SIT-12 (77.8% vs. 29.6%, *P*=0.019) or B-SITC (55.6% vs. 14.8%, *P*=0.026). There was a statistical difference between the PD and MSA using Ch-SIT-12 to test the olfactory function (*P*=0.046).

**Conclusions:**

Our results indicated that SIT-12, Ch-SIT-12 and B-SITC can be used for the detection of olfactory dysfunction in Chinese population of PD. TD subtypes may have better olfactory function than ARD subtypes. In addition, Ch-SIT-12 may be used to differentiate PD from MSA, but that should be confirmed in a larger population.

## 1. Introduction

Olfactory dysfunction is a common nonmotor symptom in Parkinson's disease (PD) with a prevalence of 50%–90%, and it may manifest more than 20 years before diagnosis of PD [1]. This may relate to pathological changes of PD. The olfactory bulb is thought to be the induction site for alpha-synuclein pathology, which later spreads through the rostral brainstem to the cerebral cortex [1, 2]. The presence of olfactory dysfunction may elevate the risk of incident PD [3]. Olfactory dysfunction may be related to PD disease progression. A recent review found that smell detection ability seems to decrease more rapidly in the early phase of PD, indicating a possible relationship with disease progression [4]. Olfactory function may be different in tremor-dominant (TD) and akinetic-rigid dominant (ARD) subtypes of PD. Previous studies found that TD subtypes may have better olfactory function than ARD subtypes [5]. Olfactory dysfunction is also associated with common nonmotor manifestations of PD, such as apathy and cognitive impairment [6, 7]. Solla et al. [8] found strict association between global olfactory impairment and executive function deficits in PD patients. The olfactory test was important not only in PD, but also in atypical Parkinsonism. The olfactory test was considered to differentiate idiopathic PD from common differential diagnoses, because multiple system atrophy (MSA) and progressive supranuclear palsy (PSP) may suffer less from olfactory loss compared to PD [9].

Although several olfactory function testing methods exist, the University of Pennsylvania Smell Identification Test (UPSIT) and Sniffin' Sticks Test are widely used in many countries. Because of easy operation, Sniffin' Sticks 12-identification test (SIT-12) is widely used in the clinical olfactory test in European countries and in Chinese patients of PD too [10]. Although there is an officially adapted version for Asian populations, the cultures are different in various countries, so it may not suit for every culture. That is why there are different versions of the Sniffin' Sticks test in different countries to improve its diagnostic value [11, 12]. Similarly, some odors in SIT-12 are unfamiliar to Chinese too, such as cinnamon and cloves. In order to improve the diagnostic value of SIT-12 for Chinese, we exchanged 4 odors and modified SIT-12 to China modified version of SIT-12 test (Ch-SIT-12) [13]. In the previous study, we used Ch-SIT-12 to test the olfactory function in PD and healthy controls among Chinese and found that it could be used as a screening test for PD in Chinese. In China, a modified Brief Smell Identification Test for Chinese (B-SITC) which was familiar with Chinese population has been approved by the China's National Medical Products Administration (NMPA) for clinical use [14]. Compared to SIT-12, B-SITC is disposable and more expensive, which makes it relatively rarely used clinically.

In this study, we want to use three different olfactory function methods (SIT-12, Ch-SIT-12, and B-SITC) in PD and MSA to investigate which one is more suitable to Chinese. At the same time, we hope the SIT-12 could be modified better based on our study in the future.

## 2. Methods

### 2.1. Study Population

This study was approved by the Ethics Committee of Beijing Hospital, and each participant signed informed consent.

From January 2019 to July 2021, 36 patients with PD and 7 patients with MSA were enrolled in this study. We collected past medical history via face-to-face interview. Participants with possible olfactory-influencing factors including nose surgery, chronic sinusitis, acute upper respiratory tract infection, and other central nervous system diseases that may affect the sense of smell, such as Alzheimer's disease, and multiple sclerosis were excluded. During the clinical investigation, participants did not have COVID-19-related symptoms.

### 2.2. Clinical Profiling

We collected every participant's demography detail including gender, age, age of onset for PD, and duration via a face-to-face interview. The clinical status was evaluated by neurologists using the Unified Parkinson's Disease Rating Scale (UPDRS) and modified Hoehn–Yahr stage (range: 0–5) in patients with PD. If patients had motor complications, they were evaluated clinical status at the “on” time. Patients were classified based on established methods into TD or ARD subtypes using items from the UPDRS part III. The tremor score was calculated from the sum of UPDRS items 20 and 21 divided by 7. The nontremor score was calculated from the sum of UPDRS items 18, 19, 22, 27, 28, 29, 30, and 31, divided by 12. PD patients were grouped in the TD subtype if the tremor/nontremor score ratio was equal to or greater than 1 and in the ARD subtype if the tremor/nontremor score ratio was less than 1. [15].

### 2.3. Olfactory-Testing Methods

All participants underwent 3 olfactory tests, including SIT-12, Ch-SIT-12, and B-SITC.

Cinnamon, liquorice, colophony, and cloves in SIT-12 were replaced with sesame oil, soy sauce, chocolate, and garlic. The original eight unchanged odors in SIT-12 and four replaced odors combined to the Ch-SIT-12. The decision which odors were changed and which odors were chosen as replacement is based on the study by Shu et al. [16]. In order to facilitate test and reduce test time, SIT-12 and Ch-SIT-12 are integrated into a single detection, which adds 4 modified odors to the original 12 odors, resulting in 16 odors. We did not tell the participants whether the test had been modified.

SIT-12 and Ch-SIT-12 required the participants to refrain from eating or drinking 15 minutes before the test. The 16 felt-tip pens presenting odors were consecutively placed approximately 2 cm in front of both nostrils for 3-4s, with a 30 s interval between odor presentations. For each odor, the participants were forced to choose the correct odor from a list of 4 descriptors. Each correct answer received a score of 1. The scores of SIT-12 and Ch-SIT-12 were calculated, respectively, the identification score ranges from 0 to 12, and the cutoff value was set at 8.

B-SITC required the participants to flex the pack three times and then open it from one side. The participants were then presented with an odor block for a few seconds before choosing one of four available responses from the answer sheet. The interval between each odor was limited to 15s and the total time to complete the test was less than 5 minutes. The number of correct answers in relation to the 12 odors was summed and the cutoff value was set at 8.

### 2.4. Statistical

The paired Chi-square test (McNemar test) was used to compare the differences between the three olfactory tests in patients with PD. Fisher's exact test was used to compare the olfactory differences between TD and ARD subtypes. Pearson chi-square or Fisher's exact test was used to compare the olfactory differences between patients with PD and MSA using different olfactory tests.

## 3. Results

### 3.1. The Differences of Three Olfactory Tests in Patients with PD

36 patients with PD including 23 males (63.9%) and 13 females (36.1%) were enrolled. The average age and duration of disease are shown in Table 1. The UPDRS-I score was 2.3 ± 2.63, UPDRS-II score was 9.2 ± 6.47, and the UPDRS-III score was 25.5 ± 11.22. The paired Chi-square test was used to compare the difference between three groups. There was no significant difference between three olfactory tests in patients with PD (B-SITC vs. SIT-12: *P*=0.508; Ch-SIT-12 vs. B-SITC: *P*=0.146; and SIT-12 vs. Ch-SIT-12: *P*=0.375).

TD subtypes have higher percentage of normal olfactory function than ARD subtypes in all three olfactory tests. Using Ch-SIT-12 or B-SITC, there was significant difference between TD and ARD subtypes (Table 2).

### 3.2. The Differences of Three Olfactory Tests in Patients with PD and MSA

Except for patients with PD, there are 7 patients with MSA were enrolled. The demography details of MSA are shown in Table 1. There were no differences in gender and disease duration between the two groups. Patients with MSA were younger than patients with PD. According to the cutoff value, all participants were classified as normal olfactory function or olfactory dysfunction using three olfactory testing methods. We found the percentage of normal olfactory function was higher in MSA compare to PD, no matter which olfactory testing method was used. However, it was only using Ch-SIT-12 that there was a statistical difference between the PD and MSA (*P*=0.046). No difference between the two groups using B-SITC (*P*=0.378) or SIT-12(*P*=0.394).

### 3.3. The Difference of Modified Odors in Ch-SIT-12 and Original Odors in SIT-12

**Figure 1:**
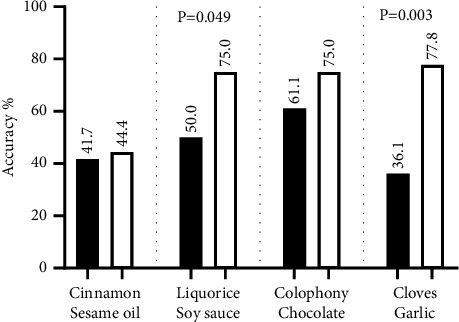
Accuracy of the four modified odors compared with four original odors in PD.

Patients with PD had higher accuracy of the four modified odors compared with four original odors and there are significant differences in liquorice-soy sauce groups (*P*=0.049) and cloves-garlic groups (*P*=0.003) (Figure 1).

**Figure 2:**
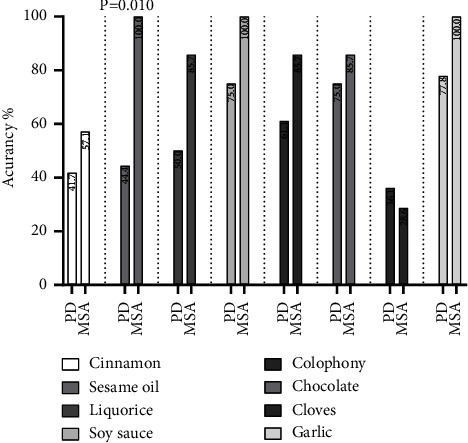
Comparing the accuracy of 8 odors between PD and MSA.

Patients with MSA also had higher accuracy of the four modified odors compared with four original odors and even has 100% accuracy in three modified odors. However, only the accuracy of sesame oil odor has difference between PD and MSA (44.4% vs. 100%, *P*=0.010). The accuracy of other three modified odors has no difference between PD and MSA (Figure 2).

## 4. Discussion

The olfactory tests for Parkinson's disease including UPSIT and Sniffin' Sticks test were developed and widely used in western countries. However, the sensitivity and specificity were less analyzed in Chinese population. Among a few studies of olfactory function tests in China, they [17, 18] have studied the olfactory detection of Sniffin' Sticks test in Chinese population, suggesting that it can distinguish PD patients from normal people. Due to cultural differences, some of its odors, such as cloves and cinnamon, are not familiar with Chinese people [13], which may result in inaccurate results. In China, B-SITC was familiar with Chinses population, but it is disposable and expensive. Therefore, we investigate whether Ch-SIT-12 could be more suitable for Chinese people and reduce the cost of olfactory detection.

Our previous research has shown that Ch-SIT-12 can discriminate Chinese PD patients from controls with a sensitivity of 59% and specificity of 97% [13]. Through this study, we found that there was no significant difference between SIT-12, Ch-SIT-12, and B-SITC in PD, suggesting that the three olfactory testing methods can all be used for olfactory detection of Chinese PD. Previous studies have shown that many methods can be used for olfactory detection in Chinese PD patients. The Chinese smell identification test which was developed specifically for Chinese populations had a sensitivity and specificity of 71.1% and 89.3% for the detection of Parkinson's disease [19]. The brief smell identification test had a sensitivity of 64.1% and specificity of 83.9% for identifying PD [20]. The sensitivity and specificity of the UPSIT for the diagnosis of olfactory dysfunction in early Taiwanese PD was 86% and 70% [21].

Few specific studies evaluated the differences in olfactory function between the TD and ARD subtypes, but the olfactory function in different PD subtypes is still not clearly known [5, 22, 23]. We found that TD subtypes may have relatively better olfactory function than ARD subtypes, which was similar to some previous studies [5, 22, 23]. However, we need to further expand the sample size to verify the result.

We also found using Ch-SIT-12 that there was a statistical difference between the PD and MSA, but using SIT-12 and B-SITC did not find this. Few studies focused on the olfactory function of MSA and PD, and none has been found in Chinese patients. Previous reports indicated that the olfactory function is relatively intact or slightly reduced in patients with MSA [24]. Some olfactory tests may be useful in the differential diagnosis of early-stage PD from MSA [9]. Fujita et al. [25] found that the sensitivity and specificity of differentiating PD and atypical parkinsonian syndromes were 58.4% and 76.3% by card-type odor identification testing. Krismer [26] found using Sniffin' Sticks test that battery separating PD from MSA was 76.7% (sensitivity) and 95.7% (specificity). It is not clear why olfactory dysfunction is rather slightly in MSA compared to PD. Chen et al. [27] found that compared to healthy controls and MSA patients, the volumes of the olfactory bulb and tract were significantly reduced in PD patients, which reflects a difference in pathology of PD and MSA.

We found that the accuracy of modified odors was higher than the original odors in PD, especially licorice and clove. In addition, the accuracy of sesame oil odor has difference between PD and MSA. Our previous study also found that cinnamon and clove were recognized by less than 75% of the Chinese group of healthy controls. We think the modified odors of cinnamon, licorice, and clove are meaningful. The replacement of colophony to chocolate may be meaningfulness and need to improve further.

This study has many limitations. First, the number of participants was too small, especially for MSA, which may lead to the inaccurate results. Second, we did not distinguish the types of MSA, such as MSA-P and MSA-C. Finally, normal olfactory ability should be considered before inclusion of patients.

In the future, we can include more participants, especially patients of MSA. We can modify the odors to be more suitable to Chinese based on our result and record the olfactory ability before inclusion of participants.

## 5. Conclusion

We made a Chinese version of SIT-12 by modifying four odors in SIT-12. The results of Ch-SIT-12, SIT-12, and B-SITC in detecting olfactory dysfunction in Chinese PD patients are similar, suggesting that these three olfactory testing methods can all be used for the detection of olfactory dysfunction in PD. TD subtypes may have relatively better olfactory function than ARD subtypes. In addition, Ch-SIT-12 may be used to differentiate PD from MSA. However, these are preliminary data that should be confirmed in a larger population. We may provide a new olfactory testing method for Parkinsonism of Chinese and we hope to further modify SIT-12 to be more suitable for the Chinese population in future studies.

## Figures and Tables

**Table 1:** Demographic data and olfactory tests results of PD and MSA.

Items	Patients with PD (*n* = 36)	Patients with MSA (*n* = 7)	*p* value
Gender (male %)	23 (63.9%)	4 (57.1%)	1.000
Age (year)	65.6 ± 12.15	57.1 ± 7.63	0.034^*∗*^
Duration (month)	100.3 ± 134.51	51.4 ± 37.82	0.485
B-SITC (normal olfactory function)	9 (25%)	3 (42.8%)	0.378
Ch-SIT-12 (normal olfactory function)	15 (41.6%)	6 (85.7%)	0.046^*∗*^
SIT-12 (normal olfactory function)	12 (33.3%)	4 (57.1%)	0.394

^*∗*^ *P* < 0.05.

**Table 2:** Three olfactory tests results of TD and ADR subtypes.

Items	TD subtypes (*n* = 9)	ADR subtypes (*n* = 27)	*p* value
Gender (male %)	3 (33.3%)	20 (74.1%)	0.046^*∗*^
Age (year)	63.7 ± 6.02	66.2 ± 13.63	0.597
Duration (month)	113.5 ± 110.97	95.3 ± 145.37	0.302
B-SITC (normal olfactory function)	5 (55.6%)	4 (14.8%)	0.026^*∗*^
Ch-SIT-12 (normal olfactory function)	7 (77.8%)	8 (29.6%)	0.019^*∗*^
SIT-12 (normal olfactory function)	5 (55.6%)	7 (25.9%)	0.126

^*∗*^ *P* < 0.05.

## Data Availability

The data used to support the findings of this study are available on request from the corresponding author.
